# Supplementary material for: Protective Effect of Follicle-Stimulating Hormone on DNA Damage of Chicken Follicular Granulosa Cells by Inhibiting CHK2/p53
Source: Cells. 2022 Apr 11;11(8):1291. doi: 10.3390/cells11081291 (PMC9031212; doi:10.3390/cells11081291)
Supplement: Supplementary file 1 [file cells-11-01291-s001.zip › cells-1623767-supplementary.pdf]

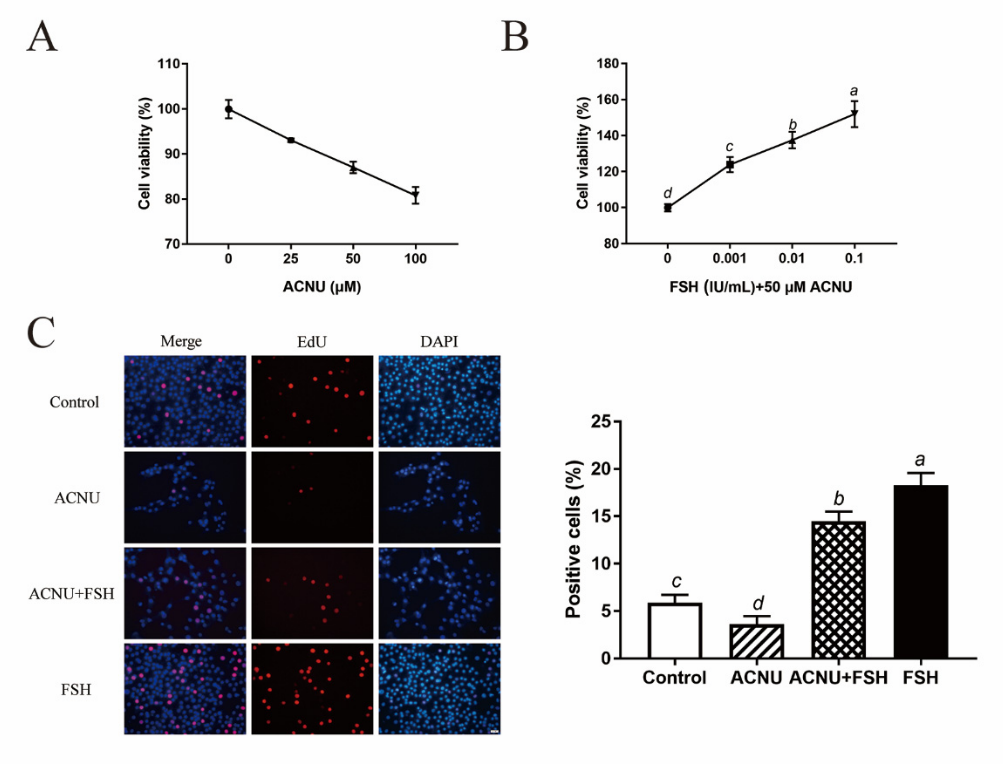

Figure S1. Effects of ACNU or FSH on viability and proliferation of GCs via EdU incorporation. Values are the means ± SEM (n = 3). Bars with different superscripts are statistically different ($p < 0.05$). (**A**) Cell viability of GCs after treatment with ACNU. (**B**) Viability of GCs after treatment with ACNU and FSH. (**C**) Alleviating effect of FSH on the decreased proliferation of GCs treated with ACNU. Scale bar: 20 μm. Bars with different superscripts are statistically different ($p < 0.05$).
